# Structure determination of an 11-subunit exosome in complex with RNA by molecular replacement

**DOI:** 10.1107/S0907444913011438

**Published:** 2013-10-12

**Authors:** Debora Lika Makino, Elena Conti

**Affiliations:** a Structural Cell Biology, Max Planck Institute of Biochemistry, Am Klopferspitz 18, 82152 Martinsried, Germany

**Keywords:** exosome, molecular replacement, model building, RNA, nucleases, Rrp44

## Abstract

The RNA exosome is an evolutionarily conserved multi-protein complex involved in the 3′ degradation of a variety of RNA transcripts. In the nucleus, the exosome participates in the maturation of structured RNAs, in the surveillance of pre-mRNAs and in the decay of a variety of noncoding transcripts. In the cytoplasm, the exosome degrades mRNAs in constitutive and regulated turnover pathways. Several structures of subcomplexes of eukaryotic exosomes or related prokaryotic exosome-like complexes are known, but how the complete assembly is organized to fulfil processive RNA degradation has been unclear. An atomic snapshot of a *Saccharomyces cerevisiae* 420 kDa exosome complex bound to an RNA substrate in the pre-cleavage state of a hydrolytic reaction has been determined. Here, the crystallographic steps towards the structural elucidation, which was carried out by molecular replacement, are presented.

## Abstract 2

The crystallographic steps towards the structure determination of a complete eukaryotic exosome complex bound to RNA are presented. Phasing of this 11-protein subunit complex was carried out *via* molecular replacement.

## 1. Introduction   

**Figure 1:**
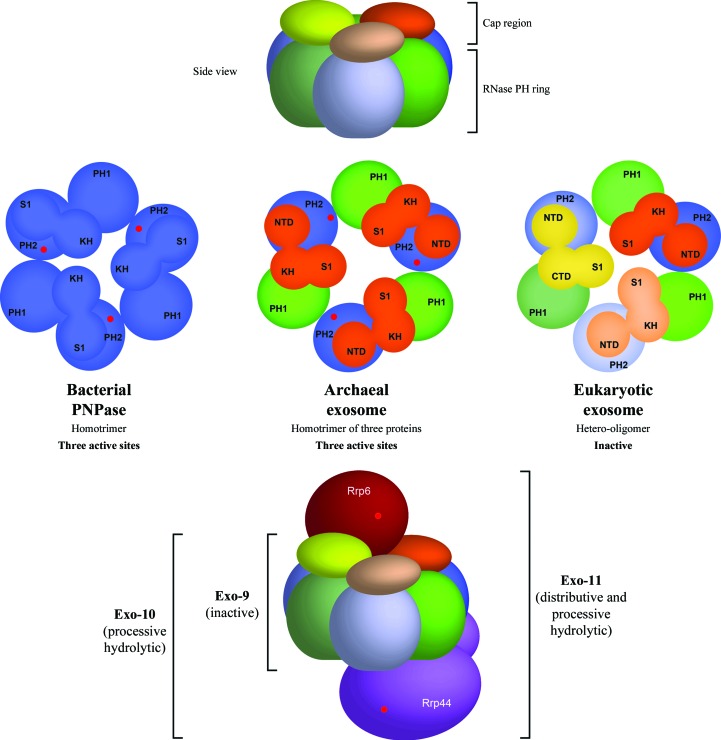
Structural organization of RNase PH complexes. The top panel shows a side view of their ring arrangement, with the S1/KH domains, also called the cap region, on top. The middle panel illustrates side-by-side the evolutionary architectural conservation of the RNase PH complexes. In bacterial PNPase, one chain contains two RNase PH domains and one S1/KH region, forming a homotrimer with three phosphorolytic active sites. The archaeal exosome evolved into three distinct subunits, carrying RNase PH subunits, Rrp41 and Rrp42, and a cap protein, which could be either Rrp4 or Csl4. This complex comprises a homotrimer of three different proteins that, similarly to the bacterial PNPase, has three phosphorolytic sites. The eukaryotic exosome, however, is composed of nine different subunits that are still somewhat related in sequence to the archaeal Rrp41-like subunits (Rrp41, Rrp46 and Mtr3), the archaeal Rrp42-like subunits (Rrp45, Rrp43 and Rrp42) and the cap proteins (Rrp4, Csl4 and Rrp40). As a consequence of this increase in structural complexity, the eukaryotic exosome core is catalytically inactive. Its catalytic function arises from the association of a tenth subunit, Rrp44 (violet; bottom panel), a processive hydrolytic exoribonuclease. In the nucleus of yeast cells, an eleventh component, Rrp6 (red; bottom panel), binds to the exosome, providing a second exoribonucleolytic site to the entire complex.

The eukaryotic exosome core, Exo-9, contains six RNase PH-like subunits that assemble into a ring-like structure and three proteins composed of S1/KH domains (so-called cap proteins) forming a coaxial ring (Fig. 1 ▶, top; Mitchell *et al.*, 1997 ▶). Overall, Exo-9 has a barrel-like structure with a prominent central channel. This architecture is evolutionarily conserved, sharing significant structural similarity with archaeal exosomes and bacterial PNPase (Lykke-Andersen *et al.*, 2009 ▶). However, the complexity of subunit composition and of catalytic activity of Exo-9 changes from prokaryotes to eukaryotes (Fig. 1 ▶, bottom). In bacteria, PNPase consists of three identical proteins, each containing two RNase PH domains and an S1/KH domain in a single polypeptide chain (Symmons *et al.*, 2000 ▶). This homotrimeric complex shows a very similar domain organization to Exo-9. In archaea, two distinct proteins (Rrp41 and Rrp42) with an RNase PH fold and a third protein (Rrp4 or Csl4) with S1/KH domains also trimerize into an Exo-9-like architecture (Büttner *et al.*, 2005 ▶; Lorentzen *et al.*, 2005 ▶; Navarro *et al.*, 2008 ▶). Both complexes present phosphorolytic ribonuclease activities provided by one of the RNase PH subunits. These prokaryotic Exo-9-like complexes have three active sites owing to their homotrimeric organization. The active sites are located in a cavity shielded from exterior solvent and reachable from the central channel of the barrel. On the other hand, in the eukaryotic exosome nine different proteins provide the six RNase PH-like units and the three S1/KH units. Remarkably, as an evolutionary result of amino-acid substitutions in the active site, all eukaryotic RNase PH subunits have lost their nuclease activity, giving rise to a catalytically inactive Exo-9. In yeast and humans, the nuclease activity arises from the association of a tenth subunit, Rrp44, a processive hydrolytic exoribo­nuclease (Dziembowski *et al.*, 2007 ▶; Liu *et al.*, 2006 ▶). This complex, Exo-10, is ubiquitously present in both the nucleus and cytoplasm of eukaryotic cells. In the yeast nucleus, Exo-10 associates with an eleventh protein, Rrp6, which harbours a distributive exoribonuclease activity (Briggs *et al.*, 1998 ▶).

Previous structural work elucidated the architecture of Exo-­9 from humans (Liu *et al.*, 2006 ▶). A first view of how the processive nuclease might bind Exo-9 was later provided by the structure of Rrp44 bound to two RNase PH-like proteins of the yeast exosome (Rrp41 and Rrp45; Bonneau *et al.*, 2009 ▶). Superposition of these two structures allowed the generation of a pseudo-atomic model of Exo-10 that could be fitted into the corresponding EM reconstruction (Wang *et al.*, 2007 ▶; Malet *et al.*, 2010 ▶). Biochemical data suggested the presence of a long RNA-binding path traversing the internal channel of the barrel. However, it has not been possible to extrapolate from the pseudo-atomic model a substrate path that would explain the biochemical data. We therefore set out to crystallize and determine the structure of the complete exosome complex bound to RNA (Makino, Baumgärtner *et al.*, 2013 ▶). Here, we present the steps leading to the structure determination.

## 2. Sample preparation of a multi-subunit exosome complex   

To crystallize the *Saccharomyces cerevisiae* exosome complex, we expressed all 11 subunits recombinantly in *Escherichia coli* (either as single proteins or as binary subcomplexes). We then reconstituted the complex *in vitro* using protocols similar to those previously reported (Greimann & Lima, 2008 ▶). A critical step in the crystallization of multi-protein complexes is to ensure the chemical and conformational homogeneity of the sample. A typical problem is the chemical heterogeneity owing to the presence of subcomplexes that arise from the reconstitution procedure. This type of contamination can be problematic for crystal nucleation, growth and lattice order. A strategy often used to overcome this problem is to add an affinity tag to the least expressed or to the most labile subunit in the complex. For example, the addition of an uncleavable C-­terminal polyhistidine tag to gp62 in the gp62–gp44 clamp-loader complex was crucial to remove the tetrameric gp44 species from the sample and allowed the successful growth of T4 clamp-loader crystals (Kelch *et al.*, 2011 ▶). Another strategy is to use high-resolution ion-exchange columns, provided that the complex is stable under salt concentrations higher than the physiological value. With a shallow salt gradient, sub­complexes can be separated from the holo complex based on surface-charge differences created by the absence of one or more components in the complex. In the case of the yeast exosome, a final high-resolution ion-exchange purification step was critical to remove Exo-9 subcomplexes (Greimann & Lima, 2008 ▶) and to remove nucleic acid-bound Rrp44 species from the apo Rrp44 subunit (Makino, Baumgärtner *et al.*, 2013 ▶).

To crystallize a nuclease complexed to an RNA substrate, the enzyme has to be inactivated by mutations that abolish catalytic activity without impairing substrate binding. These nuclease mutants often carry endogenous nucleic acids from the expression in bacteria throughout purification (Frazão *et al.*, 2006 ▶). The removal of nucleic acids from the protein or complex is a fundamental step to form an apo complex that can be subsequently screened for crystallization in the presence of different RNA substrates. This step can be monitored by assessing the *A*_260_/*A*_280_ ratio of the sample. In the case of the exosome, two single-site mutations inactivated the Rrp44 nuclease (Lebreton *et al.*, 2008 ▶; Schaeffer *et al.*, 2009 ▶; Schneider *et al.*, 2009 ▶; Dziembowski *et al.*, 2007 ▶). Use of a high-resolution ion-exchange column allowed the separation of a peak at lower salt concentrations (*A*_260_/*A*_280_ ratio of ∼0.55) from a peak that eluted at higher salt concentrations (*A*_260_/*A*_280_ value of ∼0.8 or higher) and contained RNA.

## 3. Crystallization   

Screening Exo-10 sample preparations with different RNA substrates failed to yield crystals. There are various reasons why a chemically homogeneous sample may not crystallize. Firstly, the sample might dissociate or become unstable. For example, *Panicum mosaic virus* crystals only grew after keeping the sample under acidic conditions throughout all purification and crystallization steps to increase the stability of the capsid shell (Makino, Larson *et al.*, 2013 ▶). Secondly, the proteins might have surface properties that are not amenable to the formation of crystal contacts. A strategy often used is to change species (see, for example, Murachelli *et al.*, 2012 ▶). With conserved proteins, the structural core and important functional sites are usually conserved, while surface residues that are not important for function diverge, with the exception of some specific proteins such as immunoglobulins. As surface residues mediate crystal contacts, changing orthologues often changes the crystallization properties of the sample. With multi-protein complexes, however, this is clearly not an appealing strategy. The third, and perhaps the most common problem, is conformational heterogeneity arising from the presence of unstructured regions or flexible domains. In the case of the exosome, removing conformational heterogeneity was key to obtaining crystals. One subunit (Csl4) was known from EM studies to be unstable (Wang *et al.*, 2007 ▶). We first deleted this subunit and tested biochemically that the Exo-10-ΔCsl4 complex retained RNA-binding properties (Malet *et al.*, 2010 ▶). This complex did yield crystals; however, they never diffracted beyond 8 Å resolution. We then proceeded to biochemically verify whether an additional subunit might stabilize Csl4. We identified such a subunit in the eleventh exosome component, Rrp6, and mapped the stabilizing effect to a C-terminal region which essentially shows no sequence conservation and is predicted to be unstructured. The Exo-10-Rrp6_C-term_ complex crystallized and diffracted to 2.8 Å resolution in the presence of an RNA that we designed based on knowledge from biochemical assays (a 5′ duplex linked by a tetra-loop and a long 3′ poly-U_31_ overhang).

## 4. Data collection and processing   

**Figure 2:**
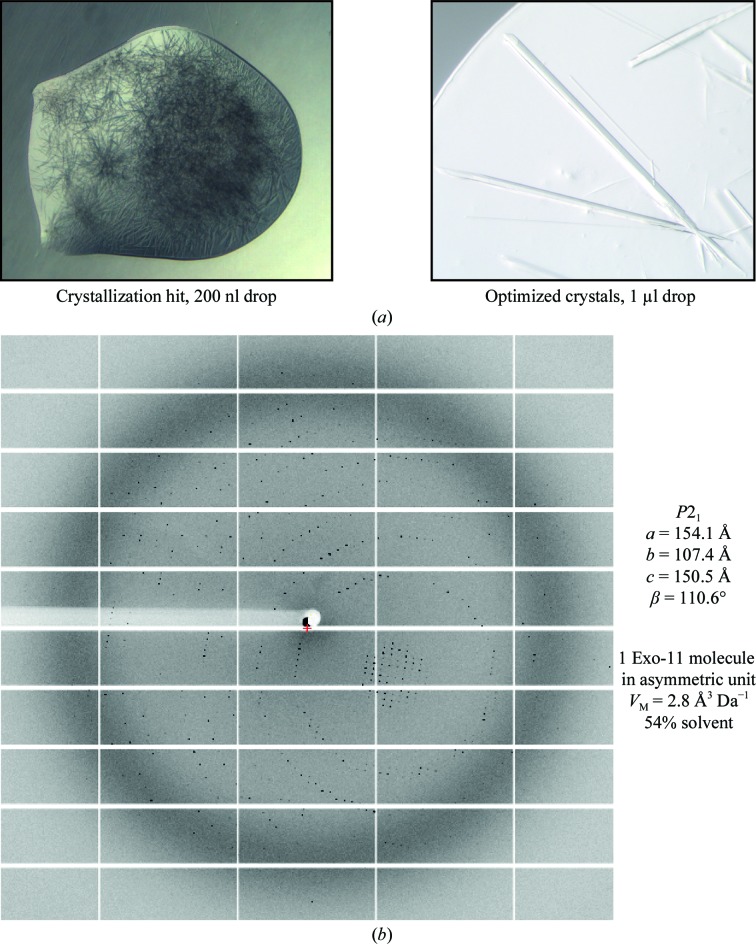
Exo-10-Rrp6_C-term_–RNA crystals and diffraction pattern. (*a*) Exosome crystals were obtained in a condition from the Qiagen PACT screen (left panel) and were optimized in Cryschem plates (right panel), where crystals grew to completion within two weeks at 292 K. The largest crystals measured 20 × 50 × 2500 µm. (*b*) Diffraction pattern recorded using a PILATUS 6M detector (Dectris) at the X10SA beamline at the Swiss Light Source, Villigen, Switzerland. Data were indexed in a monoclinic unit cell with a Matthews coefficient of 2.8 Å^3^ Da^−1^ containing one complex in the asymmetric unit.

Crystals of Exo-10-Rrp6_C-term_–RNA grew with a needle-like morphology in an optimized condition consisting of 11.4–12.2%(*w*/*v*) PEG 3350, 0.27 *M* NaBr, 0.10–0.15 *M* MES pH 6.5 (Fig. 2 ▶ *a*). To obtain reflections to 2.8 Å resolution, it was crucial to identify a suitable cryoprotectant for this crystal. A screen of several cryogenic conditions identified a mixture of a higher PEG concentration [25%(*w*/*v*)] and small amounts of glycerol [10%(*v*/*v*)] as the best cryoprotectant. As has previously been suggested, it is possible that high concentrations of glycerol create disorder upon diffusion through solvent channels and that a better solution is a mixture of small cryogenic molecules (which can immediately diffuse through solvent channels) and a large cryogenic molecule (which cannot easily enter solvent channels) (Kriminski *et al.*, 2002 ▶). With hindsight, it is also possible that the higher PEG concentration might have resulted in dehydration and crystal stabilization.

The exosome crystals were rapidly affected by radiation damage. The problem of data collection was also compounded by the fact that the crystals belonged to a monoclinic space group, which requires relatively wide reciprocal-space coverage. Over 160 segments of data were collected in order to obtain data to the highest resolution and completeness as possible. The availability of sensitive detectors with an ultra-fast readout capability certainly contributed to successful data collection. Each image was analyzed for resolution decay owing to radiation damage, for the region of the reciprocal space covered and for the feasibility of merging with other sub-data sets. The final statistics are the result of combining 46 data fragments. Most crystals diffracted to around 3.2 Å resolution, and data were obtained to 2.8 Å resolution at some specific locations on two needle crystals (Fig. 2 ▶ *b*). All data were processed using *XDS* and were merged and scaled in *XSCALE* (Kabsch, 2010 ▶).

## 5. Molecular replacement   

**Figure 3:**
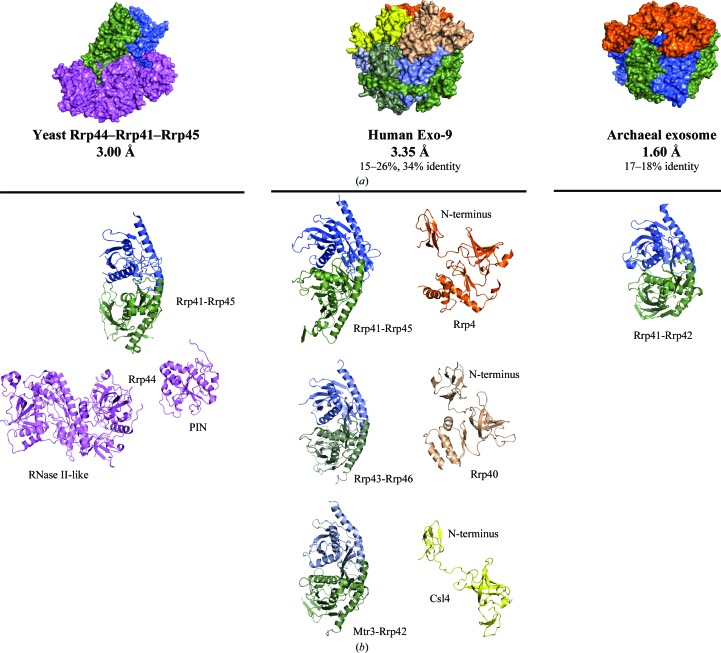
Search models used in molecular replacement. (*a*) Yeast Rrp44–Rrp41–Rrp45 ternary complex (PDB entry 2wp8; Bonneau *et al.*, 2009 ▶), human Exo-9 (PDB entry 2nn6; Liu *et al.*, 2006 ▶) and archaeal exosome (PDB entry 2je6; Lorentzen *et al.*, 2007 ▶). Although the architecture of the RNase PH barrel is evolutionarily conserved, these structures were not good enough for MR searches. (*b*) MR became more promising on disassembling the complexes into individual subunits (RNase PH pairs) and breaking the Rrp44 chain into the PIN domain and the RNase II-like region. A C-terminal region of the yeast Rrp40 structure (PDB entry 2ja9; Oddone *et al.*, 2007 ▶), was used as search model instead of the human Rrp40.

The entire molecular-replacement process was performed with *Phaser* (McCoy *et al.*, 2007 ▶) as implemented in the *CCP*4 package (Winn *et al.*, 2011 ▶); model building was performed with *Coot* (Emsley & Cowtan, 2004 ▶) and refinement was performed with *PHENIX* (Adams *et al.*, 2010 ▶). X-ray structures of several eukaryotic and prokaryotic exosome subcomplexes are available. We selected the most closely related structures based on sequence as molecular-replacement (MR) search models, namely the human Exo-9 complex (3.35 Å resolution; *R*_free_ of 34.4%; Liu *et al.*, 2006 ▶), the yeast Rrp44–Rrp41–Rrp45 ternary complex (3.0 Å resolution; *R*_free_ of 26.3%; Bonneau *et al.*, 2009 ▶; Fig. 3 ▶ *a*) and the yeast C-­terminal Rrp40 (2.2 Å resolution; *R*_free_ of 20.6%; Oddone *et al.*, 2007 ▶). While the six RNase PH subunits of Exo-9 are known to have a rather rigid fold, the three cap proteins (Csl4, Rrp4 and Rrp40) and the nuclease (Rrp44) are multidomain proteins with the expected conformational variability. The cap proteins have S1/KH domains and rather separate N-terminal domains (Fig. 3 ▶ *b*). Rrp44 has an RNase II-like exoribonuclease region (formed by a catalytic domain and three OB-fold domains) and a separate N-­terminal PIN domain (Fig. 3 ▶ *b*). Solution searches using these complexes as a whole failed, including a search for the Exo-9 barrel in the absence of the cap proteins (Fig. 3 ▶ *a*). We subsequently divided the complexes into RNase PH pairs (Rrp41-Rrp45, Rrp42-Mtr3 and Rrp43-Rrp46) and three separate cap proteins (Rrp4, Rrp40 and Csl4) and subdivided Rrp44 into the PIN domain and the RNase II-like region (Fig. 3 ▶ *b*).

The search order in the molecular replacement of this multi-protein complex proved to be important for successful phase determination by MR. We observed that subunits that are more divergent or have a small size relative to the overall complex are more easily found if some fraction of the complex has already been properly placed. Accordingly, the search order was devised to start with the evolutionarily less divergent subunits and to end with the more variable domains. Table 1 ▶ shows the output for a molecular-replacement run in the case of the exosome. As the log-likelihood gain (LLG) value calculated by *Phaser* is cumulative, the overall LLG is largely negative owing to the contribution of the last two search models, Rrp4 and Csl4. When dealing with multi-subunit complexes, a negative total LLG does not unequivocally imply an incorrect solution. The statistical values output for each subunit search, as shown in Table 1 ▶, indicated positive LLG values for the first five rounds. Inspection of the corresponding electron-density maps confirmed that these solutions were correct. The sixth search model, human Mtr3-Rrp42, yielded a slightly negative LLG value of −10. This model was correctly placed at the expected location but showed spurious density (see below). The actual problem arose with two cap proteins, Rrp4 and Csl4, for which the human orthologues were used as search models. With negative overall LLG values of −346 and −556, respectively, the MR solutions were structurally inconsistent and showed random electron-density patterns. We removed these two proteins from the search and proceeded with careful rigid-body refinement of the domains within each solution.

**Figure 4:**
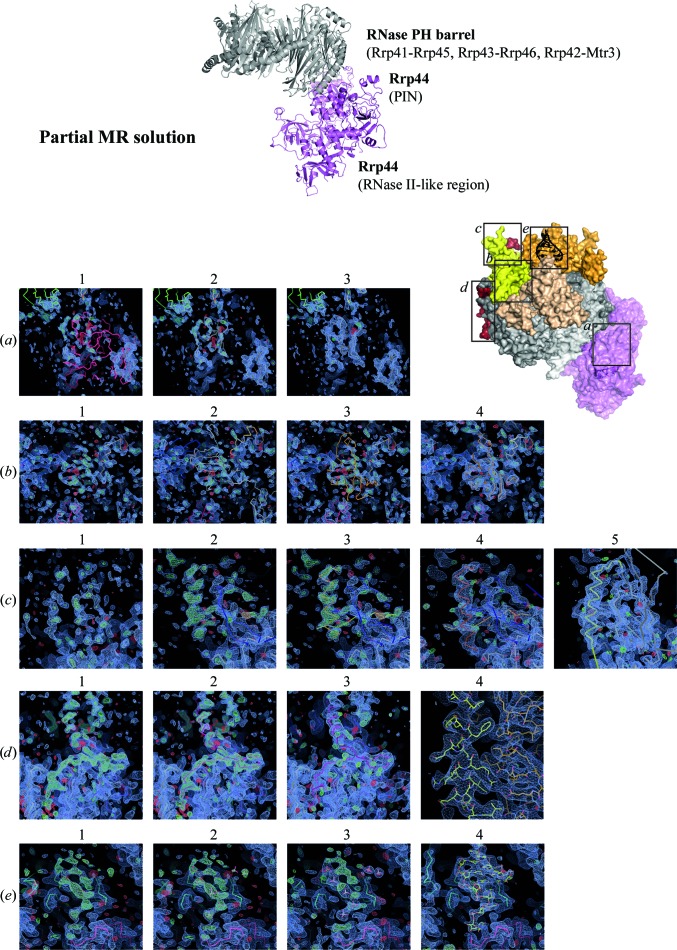
Partial MR solution comprising about 60% of the complex (top). Manual placement of missing proteins was necessary, as it was not possible to obtain solutions for these domains by MR procedures. Interpretable positive density appeared as the model improved and became more complete from (*a*) to (*e*). (*a*) The CSD1 domain position of Rrp44 was offset by 12 Å, which could not be corrected by rigid-body refinement. Manual placement to the correct position and fine adjustment by rigid-body refinement resulted in much stronger electron density for this domain. (*b*) Positive density resembling a β-­barrel was identified as belonging to the C-terminal domain of the Csl4 cap protein after superposing human Exo-9 on the partial structure. After a round of rigid-body refinement, electron density appeared with similar intensity as that of the neighbouring proteins. (*c*) The N-terminal domain of Csl4 was more difficult to discern, as the density was too large for the available model. Careful addition of backbone atoms revealed a more extended β-barrel fold than in the model. The α-helix turned out to belong to a region of the Rrp6 C-terminal tail. (*d*) Towards the end of model building, when all residues had been mutated to the yeast sequence and positional refinement had been employed, a curious density took shape on the surface of the Mtr3-Rrp43 subunits. After building a backbone and with a round of refinement, positive density for the side chain appeared. Using secondary-structure predictions of the unknown structure of the Rrp6 C-terminal tail together with good judgement of the chemical environment helped to place the side chains into the correct register. The final density for this region is shown and it indeed belonged to the C-terminal tail of Rrp6. (*e*) At the cap region, strong positive density suggested the possibility of an ordered ribonucleic acid chain. The phosphate ions placed into the strongest peaks turned out to be at distances typical of those of an RNA phosphate backbone. The electron density improved after a round of refinement, which allowed the placement of the respective ribose rings and bases. In fact, the initial positive density belonged to a strand of a duplex, as shown in the final model and the 2*mF*_o_ − *DF*_c_ map.

While the density improved for the most part, the human Mtr3-Rrp42 search model was problematic as the MR solution resulted in weak density that could not be improved by refinement. Instead of using the human structure, we used the orthologous archaeal structure as the search model for these two subunits. The *Sulfolobus solfataricus* Rrp41 and Rrp42 subunits share 17.2 and 18.4% sequence identity with yeast Mtr3 and Rrp42, respectively, which are lower values than the corresponding human Mtr3 and Rrp42 proteins (17.4 and 20.3% sequence identity, respectively; Sievers *et al.*, 2011 ▶; The UniProt Consortium, 2012 ▶). However, the archaeal structure (1.6 Å resolution, *R*_free_ of 24.9%; Lorentzen *et al.*, 2007 ▶) is at a higher resolution than the human counterpart (3.35 Å resolution; *R*_free_ of 34.4%; Liu *et al.*, 2006 ▶). Using *S. solfataricus* Rrp41-Rrp42 as a search model, the LLG significantly changed to high positive values (overall LLG of 605) and, unlike with the human search model, the electron-density maps improved upon refinement. At this point, the refined model comprised about 60% of the total number of atoms present in the asymmetric unit, with an *R*_free_ of 48.6% at 3.5 Å resolution (Fig. 4 ▶, top panel). The six RNase PH-like subunits, the Rrp44 PIN and RNase II-like domains and the Rrp40 C-­terminal domain were correctly placed. In this model, many loops for these proteins were still missing, as well as most of the cap proteins, the Rrp6 C-terminus and the RNA.

## 6. Beyond molecular replacement   

Since MR solution searches with the S1/KH domains were not successful, we started to manually position and build the missing subunits into the positive densities that became apparent in the map at this stage. For each round of model building, we restricted the refinement only to rigid body, group *B* factor and TLS, starting from a low-resolution range (typically 6–8 Å) depending on the quality of the density fit that was achieved with the model used. In the first round of refinement, all secondary structures (helices, β-sheets and β-­barrels) were divided into separate rigid groups and refined at low resolution to allow correct angular positioning of the helices relative to other secondary structures in the domain and to more accurately place β-barrels in their correct orientation. However, this approach works if the model is already rather close to the correct position and orientation. A domain of the Rrp44 RNase II-like region, CSD1, illustrates this situation. The RNase II-like region of Rrp44 as a whole was placed by *Phaser*, mainly *via* positioning of the large catalytic domain. Inspection of the electron-density map showed that the CSD1 domain, a small β-barrel domain in the RNase II-like region, was incorrectly positioned. The map showed a nearby patch of positive density with recognizable features (Fig. 4 ▶ *a*) about 12 Å away, suggesting that the CSD1 domain might assume a different conformation in the whole complex to that previously reported. Low-resolution rigid-body refinement alone could not position this domain in the density. Manual placement of the CSD1 domain into the unaccounted-for density was necessary in order for rigid-body refinement to converge and therefore to improve the density at CSD1. This step decreased the *R*_free_ value by 0.3%.

In the case of the cap proteins, we could discern electron-density features on top of the existing six RNase PH subunits where the cap proteins are expected to reside. Molecular-replacement searches had correctly found only the C-terminal S1/KH region of Rrp40; its N-terminal β-­barrel and the entire Csl4 and Rrp4 chains were still missing. To guide the identification and positioning of the cap proteins, we superposed the known human Exo-9 structure onto our current model. This procedure allowed us, for example, to identify the density for a β-­barrel on top of three RNase PH subunits, Rrp43-Rrp46-Mtr3, as the probable C-terminal domain of Csl4 (Fig. 4 ▶ *b*). Upon manual positioning and a round of refinement, the electron density for this domain improved considerably and the overall *R*_free_ value decreased by 1.1%. Other domains of the cap proteins were more difficult to identify. In particular, the human N-­terminal domain of Csl4 did not match the electron density in terms of size and β-sheet conformation. In this case, manual building of the backbone and sequence assignment was only possible at later stages of the model-building and refinement cycles, when most of the complex had been modelled with the correct sequences from yeast and several loops had been built. The final Csl4 N-terminal model is shown in Fig. 4 ▶(*c*). The root-mean-square deviation (r.m.s.d.) values (*PyMOL*; Schrödinger) between the human and the final yeast N-terminal model of Csl4 is 9.84 Å over 214 atoms, rationalizing why molecular replacement with this domain had not been successful.

## 7. Finding an unstructured subunit and the RNA   

**Figure 5:**
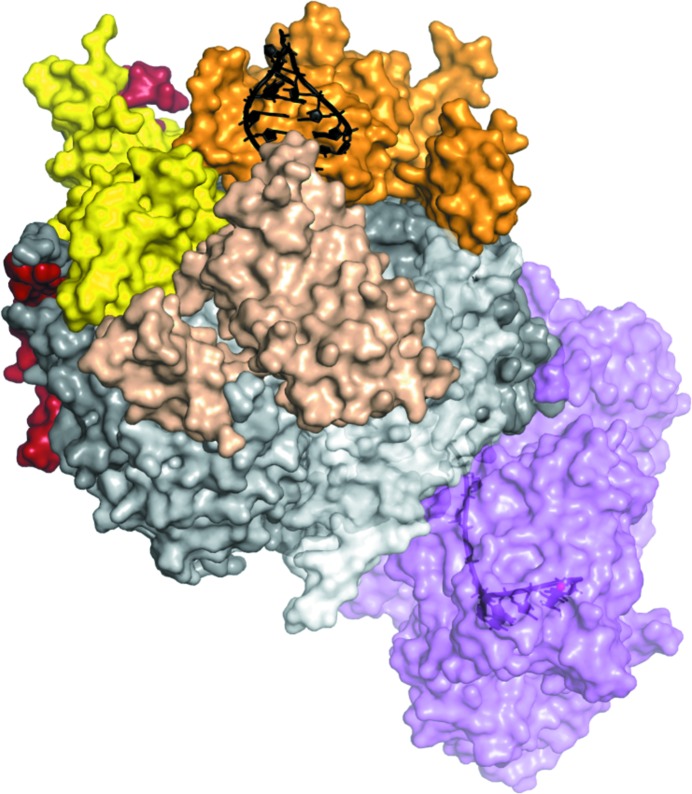
Surface representation of the final refined structure of the yeast exosome complex with the bound RNA in black (PDB entry 4ifd; Makino, Baumgärtner *et al.*, 2013 ▶). 5′ duplex RNA interacts with the cap proteins Rrp4 (orange) and Rrp40 (beige), and is in close proximity to Csl4 (yellow). The 3′ single-stranded extension passes through a central channel formed by RNase PH subunits, shown in different shades of grey. This RNA path extends into the exoribonuclease Rrp44 (violet), which is found in a closed conformation. A magnesium ion, shown as a red sphere, is found at the Rrp44 active site.

The crystals that we obtained also contained the C-terminal region of Rrp6 and an extended RNA molecule. Secondary-structure prediction of the Rrp6 C-terminal region suggested the presence of two α-helices, but the overall fold of this region was unclear. This was therefore the last protein density to be built and assigned (Fig. 4 ▶ *d*). Firstly, we built the backbone of two helices and a β-hairpin. After a round of positional and individual *B*-factor refinements, the side-chain densities became more prominent. To unambiguously assign the sequence register, we made use of information from secondary-structure predictions as well as chemical considerations based on the interacting residues on Exo-9. Eventually, when the model reached an *R*_free_ of 29.3% (2.8 Å resolution), additional electron density became apparent near the cap proteins. This density had strong peaks at regular distances typical of phosphate moieties in a nucleic acid backbone. We placed some phosphate ions into the density and, after a round of positional refinement, positive densities corresponding to riboses and bases became apparent. The final RNA duplex model and its density are shown in Fig. 4 ▶(*e*). The completed structure is presented in Fig. 5 ▶. The final model of Exo-10-Rrp6_C-term_–RNA has an *R*_free_ of 22.4% and an *R* factor of 18.3% at 2.8 Å resolution, with good stereochemistry. Overall, the model includes 26 565 non-H atoms of both protein and nucleic acid molecules refined against 99 101 unique reflections. The PDB code for this structure is 4ifd.

## 8. Discussion and conclusions   

**Figure 6:**
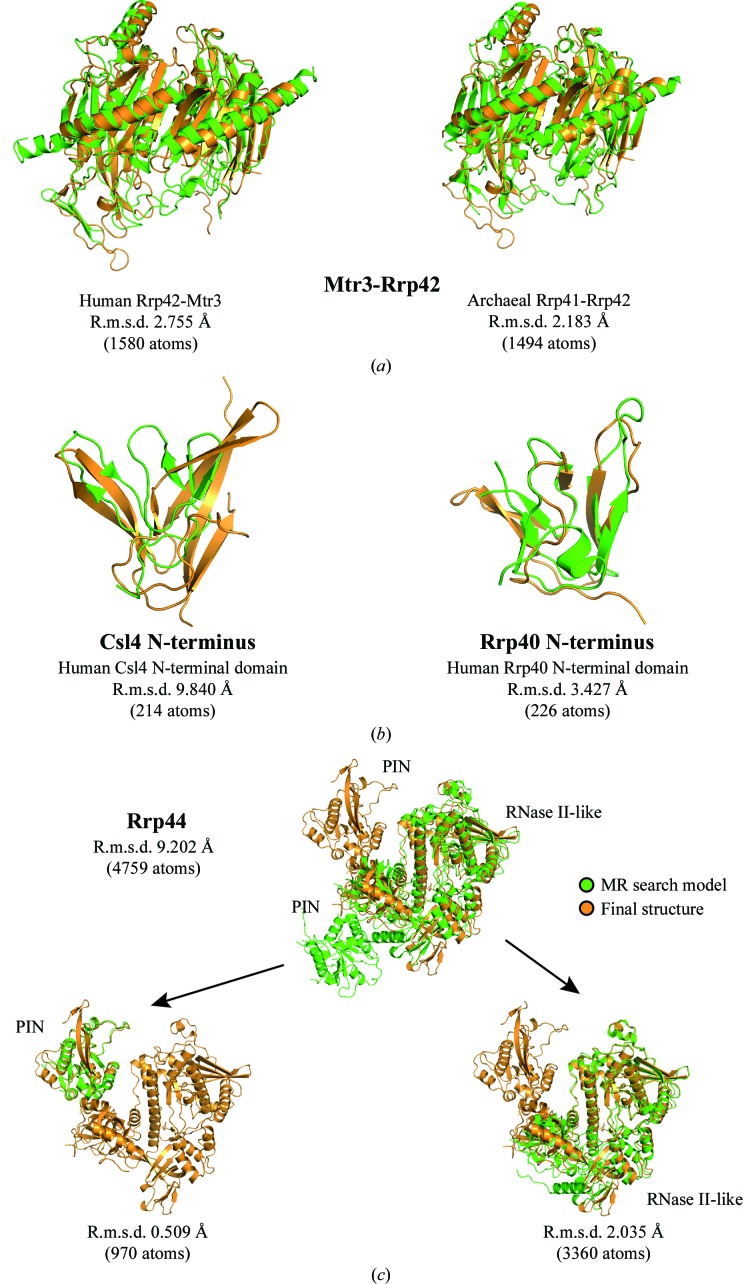
Problematic MR search models (green) and their r.m.s.d. values compared with the final structure (orange). (*a*) It was only possible to refine the Mtr3-Rrp42 solution when archaeal Rrp41-Rrp42 was used as the search model. The r.m.s.d. values are lower than those of the evolutionarily closer human Mtr3-Rrp42 model. (*b*) The N-terminal domains of Csl4 and Rrp40 were built manually. Despite their small size, their structural differences are remarkable. (*c*) Searching for the full-length Rrp44 was not possible. With an r.m.s.d. value of 9.20 Å, the conformational differences were large enough to hinder successful solution searches. Breaking the protein into two parts artificially decreases the r.m.s.d. values from 9.20 to 0.51 Å (PIN) and 2.04 Å (RNase II-like region). These two search models immediately yielded single solutions. All r.m.s.d. values were calculated using the ‘super’ command in *PyMOL* v.1.3 (Schrödinger).

Obtaining a molecular-replacement solution of this complex assembly depended not only on the quality of the processed data and resolution, but also on the quality and the tertiary-structure similarity between the search and the final models. In the case of the Mtr3-Rrp42 search model, for example, refinement and electron-density map improvement was possible when using archaeal proteins as search models, even though they are evolutionarily more distant from the yeast than the human proteins. However, the archaeal structure was at significantly higher resolution and was therefore more accurate and, with hindsight, was also more similar at the tertiary-structure level. The r.m.s.d. value between the yeast and human Mtr3-Rrp42 was 2.76 Å over 1580 atoms, whereas the r.m.s.d. with the archaeal proteins was 2.18 Å over 1494 atoms (Fig. 6 ▶ *a*). It has been noted that MR search models with r.m.s.d.s on C^α^ atoms higher than 2.5 Å can cause problems in successfully obtaining a solution (Schwarzen­bacher *et al.*, 2004 ▶). Hence, the N-terminal domains of Csl4 and Rrp40, for example, could not be phased by MR using human structures as search models, since the r.m.s.d. values between the search and final models are 9.84 Å (214 atoms) and 3.43 Å (226 atoms), respectively (Fig. 6 ▶ *b*). This corroborated the largely negative LLG contribution in the molecular-replacement solution shown in Table 1 ▶. This example could be extended to other cases in which a negative LLG could in fact have arisen from overoptimistic r.m.s.d. values between the search model and the protein in the crystal.

Changes in conformation can also hamper MR searches, as this would also result in an overall increase in r.m.s.d. values. Several exosome proteins undergo significant conformational changes when comparing the Exo-10–Rrp6_C-term_–RNA complex with subcomplexes. The cap proteins, for example, differ in their relative domain positioning as well as in their fold (Fig. 6 ▶ *b*). On the other side of the complex, the nuclease shows the most striking conformational differences. Comparison of Rrp44 in the apo form of the Rrp41–Rrp45–Rrp44 subcomplex (search model) and in the RNA-bound form of the exosome structure that we have determined results in an r.m.s.d. value of 9.20 Å over 4759 atoms. When comparing the individual Rrp44 parts, the r.m.s.d. for the N-terminal PIN domain is 0.51 Å and that for the RNase II-like region is 2.04 Å (Fig. 6 ▶ *c*). Using these separate protein regions as two independent search models (low r.m.s.d.) yielded immediate and correct MR solutions whereas the full-length protein (much higher r.m.s.d.) did not.

The structure determination of this complex posed challenging steps from reconstitution through data collection to structure determination by molecular replacement. Once stability/homogeneity of the sample and crystallization had been achieved, several issues arose: screening for a wide range of cryoprotectants, overcoming the parallax effect during crystal beam centring and dealing with very rapid resolution decay owing to radiation damage were some of the challenges that were encountered. A considerable amount of time was spent collecting and analyzing images, and high-resolution data as reported were only available at the latter stages of model building. All initial molecular-replacement trials, rigid-body and group *B*-factor refinements were performed using much lower resolution data to 3.5 Å. Higher resolution reflections were achieved later, after further extensive optimization of the crystallization and cryogen conditions, and with the availability of large number of crystals for data screening. A carefully processed data set satisfying both the completeness and resolution criteria was necessary to identify the predicted unstructured C-terminal tail of Rrp6 and to build the RNA. Finding good search models and accounting for the unknown conformational variability are also important factors to take into consideration, which, in combination with all the above factors, helped to find the path to successful molecular replacement.

## Supplementary Material

PDB reference: 11-subunit exosome in complex with RNA, 4ifd

## Figures and Tables

**Table 1:** Molecular-replacement solution scores from *Phaser* v.2.3.0 In this specific search, we used the available data to 3.5 resolution. The overall log-likelihood gain value was compromised owing to the negative contribution from search results using human cap protein models (Rrp4 and Csl4). However, the first five searches yielded correct solutions with positive LLG values. Human Rrp42-Mtr3 was also correctly placed despite the slightly negative LLG value, but subsequent refinement cycles did not improve the density. Using archaeal Rrp41-Rrp42 proteins instead, the LLG value turned out to be considerably higher than that obtained using the human proteins and the electron-density maps improved upon refinement. With the exception of the Rrp40 C-terminal region, all other cap-protein domains (the Rrp40 N-terminus and the entire Csl4 and Rrp4) had to be manually placed and built, as molecular replacement was not possible with the available structures.

Search order	RFZ	TFZ	PAK	LLG†	TFZ	Search models
1	6.8	9.7	0	168	12.6	Yeast Rrp41-Rrp45
2	4.5	13.7	0	149	15.9	Yeast Rrp44 (RNase II-like region)
3	3.8	9.8	0	277	16.8	Yeast Rrp44 (PIN domain)
4	3.8	16.1	0	450	18.3	Yeast Rrp40 C-terminal region
5	3.1	7.8	0	202	12.6	Human Rrp43-Rrp46
6	4.6	10.7	2	10	11.8	Human Rrp42-Mtr3
7	4.2	5.0	0	346	6.3	Human Rrp4 C-terminal region
8	3.7	5.5	5	556	6.6	Human Csl4 C-terminal region

† The overall LLG was 557.
